# Mechanisms of PD-1/PD-L1 expression and prognostic relevance in non-Hodgkin lymphoma: a summary of immunohistochemical studies

**DOI:** 10.18632/oncotarget.16680

**Published:** 2017-03-29

**Authors:** Pauline Gravelle, Barbara Burroni, Sarah Péricart, Cédric Rossi, Christine Bezombes, Marie Tosolini, Diane Damotte, Pierre Brousset, Jean-Jacques Fournié, Camille Laurent

**Affiliations:** ^1^ Département de Pathologie, CHU Toulouse, Institut Universitaire du Cancer de Toulouse, Centre Hospitalo-Universitaire de Toulouse, Toulouse, France; ^2^ Institut Universitaire du Cancer de Toulouse, Toulouse, France; ^3^ Centre de Recherches en Cancérologie de Toulouse, UMR1037 INSERM-Université Toulouse III, Toulouse, France; ^4^ Laboratoire d’Excellence TOUCAN, Toulouse, France; ^5^ Programme Hospitalo-Universitaire en Cancérologie CAPTOR, Toulouse, France; ^6^ Institut Carnot CALYM, Toulouse, France; ^7^ Service de Pathologie Hôpitaux Universitaires Paris Centre, Hopital Cochin, Paris, France; ^8^ CHU le Bocage, Hématologie Clinique, Dijon, France; ^9^ Centre de Recherche des Cordeliers, INSERM U1138, Paris, France; ^10^ Paul-Sabatier, ERL 5294 CNRS, Université de Toulouse, Toulouse, France

**Keywords:** PD-1/PD-L1 expression, non-Hodgkin lymphoma, prognostic value

## Abstract

Immune checkpoint blockade therapeutics, notably antibodies targeting the programmed death 1 (PD-1) receptor and its PD-L1 and PD-L2 ligands, are currently revolutionizing the treatment of cancer. For a sizeable fraction of patients with melanoma, lung, kidney and several other solid cancers, monoclonal antibodies that neutralize the interactions of the PD-1/PD-L1 complex allow the reconstitution of long-lasting antitumor immunity. In hematological malignancies this novel therapeutic strategy is far less documented, although promising clinical responses have been seen in refractory and relapsed Hodgkin lymphoma patients. This review describes our current knowledge of PD-1 and PD-L1 expression, as reported by immunohistochemical staining in both non-Hodgkin lymphoma cells and their surrounding immune cells. Here, we discuss the multiple intrinsic and extrinsic mechanisms by which both T and B cell lymphomas up-regulate the PD-1/PD-L1 axis, and review current knowledge about the prognostic significance of its immunohistochemical detection. This body of literature establishes the cell surface expression of PD-1/PD-L1 as a critical determinant for the identification of non-Hodgkin lymphoma patients eligible for immune checkpoint blockade therapies.

## INTRODUCTION

In order to develop within immunocompetent hosts, it is imperative that tumors evolve several immune escape strategies, such as mutations causing antigen loss or alteration of the antigen processing and presentation machinery [1, 2]. Other mechanisms that lead to immune evasion have also been identified in lymphoma; they include the impairment of immune cell infiltration through endothelial defects, the inhibition of immune activation by the secretion of suppressive mediators such as TGF-β and IL-10 [3], the local recruitment of immunosuppressive cells such as regulatory T cells (Tregs) [4], tumor-associated macrophages (TAMs) [5] and myeloid-derived suppressor cells (MDSC) [6, 7], and the impairment of functional anti-tumor responses through the up-regulation of immune checkpoint gene expression [8–11]. One of the most commonly dysregulated checkpoints involves the interaction of the programmed death-1 receptor (PD-1, CD279) at the surface of T lymphocytes with its ligand programmed death-ligand-1 (PD-L1) or PD-L2, which are found at the surface of macrophages and some tumor cells. This interaction delivers inhibitory signals that ultimately cause apoptosis, anergy or functional exhaustion of the T cells involved. Recently, we and others reported the over-expression of PD-L1 in non-Hodgkin lymphoma tumor cells and the increased expression of PD-1 in tumor-infiltrating lymphocytes (TILs) [9, 10, 12, 13]. Thus, treatment with immune checkpoint inhibitors targeting PD-1/PD-L1, either alone or in combination with other immune checkpoint inhibitors, can restore T cell effector function [14] and has emerged as a promising strategy for hematological malignancy therapy, particularly in patients with refractory Hodgkin lymphoma [15, 16], relapsed follicular lymphoma (FL) [17], and other aggressive non-Hodgkin lymphoma (NHL) [18, 19].

Here, we review the literature on PD-1 and PD-L1 protein expression levels in NHL and the mechanisms of their up-regulation, as well as the prognostic relevance of these proteins in NHL patients.

## THE BIOLOGY OF THE PD-1/PD-L1/2 AXIS

The main physiological role of PD-1 is in limiting autoimmunity in an inflammatory context (e.g. in response to infection) by restricting the activity of T cells in peripheral tissues [20, 21]. PD-1/PD-L1 interactions usually occur predominantly in peripheral tissues, however in some cancers, for example lymphoma developing in lymphoid organs, PD-1 engagement can reduce the anti-tumor response of effector T cells. At the intracellular level, signaling downstream of the PD-1/PD-L1/2 interaction reduces the duration of the synaptic contact [22], by down-regulating TCR signaling through a pathway thought to involve the SHP2 phosphatase [23] and thereby impairing the immunological synapse formed between effector T cells and antigen presenting cells. PD-1 is expressed by T cells, B cells and natural killer (NK) cell effectors, and has been described as an exhaustion marker in cancer and chronic viral infections [24–27]. PD-L1 (CD274) is physiologically expressed at the surface of B cells, T cells and macrophages, whereas PD-L2 (CD273) is mainly expressed by antigen-presenting cells and epithelial tissues [20]. In many solid cancers, PD-1 is upregulated by a large proportion of TILs, whereas its ligands PD-L1 and PD-L2 are expressed by a variety of tumor cells [28–30] and cause a reduction in anti-tumor immunity.

**Figure 1:**
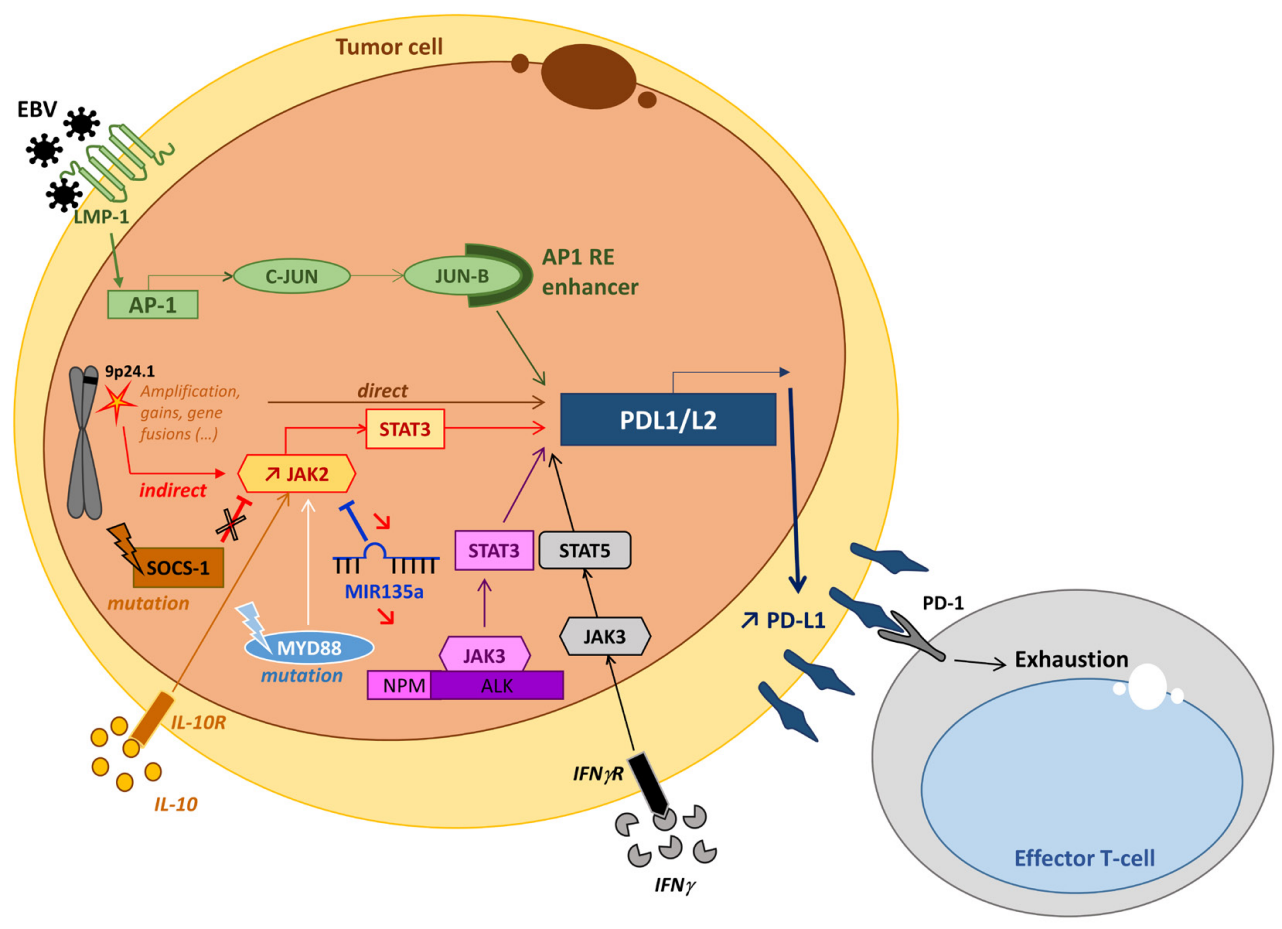
General mechanisms that lead to PD-L1 overexpression in lymphoma Genetic alterations to the PD-L1 and PD-L2 locus of chromosome 9p24.1 (gains, amplifications or fusions) directly induce the activation of the PD-L1 promoter and thus PD-L1 overexpression [35, 43]. PD-L1 expression can also be induced by activation of the JAK/STAT pathway *via* inflammatory cytokines such as IL10 [13, 39]. This is through activation of JAK2 *via* either its molecular alteration, the inhibition of SOCS-1 [36] or by microRNA miR-135a [37]. EBV infection directly activates the PD-L1 promoter *via* the AP-1/cJUN/JUN-B pathway and indirectly activates it *via* the activation of JAK3-STAT5 by inflammatory cytokines (IFN) [13, 43]. Other indirect processes that may result in molecular anomalies that induce the activation of the JAK/STAT pathway typically include the nucleophosmin-anaplastic lymphoma kinase (NPM-ALK) translocation in NPM-ALK-positive anaplastic large cell lymphoma (ALCL) [40, 41] or the MYD88 L265P mutation in diffuse large B cell lymphoma [42].

In lymphoma, PD-1 is frequently upregulated in tumor cells themselves. For example, its expression is regularly reported in peripheral T cell lymphoma (PTCL) derived from follicular helper T cells (TFH) such as angioimmunoblastic T cell lymphoma, follicular T cell lymphoma and nodal peripheral T cell lymphoma with TFH phenotype. Likewise, some neoplastic B cells (prolymphocytes/paraimmunoblasts) in chronic lymphocytic leukemia (CLL) frequently up-regulate PD-1 [28–31]. Its ligands, PD-L1 (B7-H1, CD274) and PD-L2 (B7H3, CD273), are also often expressed by the tumor cells in some B cell or T cell lymphoma (Table 1). The proliferation-reducing effect of PD-L1 blockade in different lymphoma cell lines suggests a key role for PD-1/PD-L1 expression in NHL lymphomagenesis [32]. Intracellular PD-1 signaling in effector T cells, which is activated upon binding to PD-L1 or PD-L2, reduces T cell activation signaling and inhibits efficient antitumor immune function [33]. In the microenvironment of NHL tumors, PD-1 and PD-L1 can be expressed on effector T and myeloid cells, respectively [33, 34], and participate in NHL immune escape strategies. Several mechanisms collectively referred to as intrinsic and adaptive immune resistance can account for the overexpression of PD-L1 and PD-L2 by malignant lymphoid cells. These mechanisms, schematically depicted in Figure 1, are not mutually exclusive and may co-exist in the same tumor [35–43].

**Table 1:** Summary of studies assessing PD-1/PD-L1 protein expression in NHL and its impact on NHL patient outcome

PD-L1 protein expression in NHL						
NHL subtype	Study (number of cases)	Technical approach (Antibody source / clone)	Scoring methodology -- Cut offs		Frequency of PD-L1 protein expression	Outcome
			Tumor cells	ME cells		
DLBCL	Kiyasu J. & al.,[54] *Blood* 2015 (n=1121)	PD-L1 IHC FFPE (Abcam / EPR1161)	PD-L1+ if >30% cells showed positive staining	mPD-L1+ if in >20% of the ME (tissue area)	10.5% of DLBCL had PD-L1+ tumor cells 15.3% of DLBCL had mPD-L1+ ME cells	PD-L1+ DLBCL had lower OS than PD-L1- DLBCL (50% vs 75% at 2 years, p=0.0009) (maintained in multivariate analysis with Adj. *p=0.0323*) No significant difference in OS between mPD-L1+ and mPD-L1^-^ DLBCL (*p=0.31*)
	Chen B. & al.,[13] *CCR* 2013 (n=66 DLBCL NOS and 9 DLBCL EBV^+^)	PD-L1 IHC FFPE (Sino Bio / 15)	PD-L1+ if >5% cells had a 2+ or 3+ level of staining intensity	mPD-L1+ if >20% of the ME (tissue area) had a 2+ or 3+ level of staining intensity	11% of DLBCL NOS had PD-L1+ tumor cells 14% of DLBCL NOS had mPD-L1+ ME cells 100% of EBV+ DLBCL had PD-L1+ tumor cells 100% of EBV+ DLBCL had mPD-L1+ ME cells	NA
	Laurent C. & al.,[10] *Oncoimmunol*. 2015 (n=27)	PD-L1 IHC FFPE (Ventana / SP142)	PD-L1+ if >10% cells had a 2+ or 3+ level of staining intensity	mPD-L1+ if >10% cells had a 2+ or 3+ level of staining intensity	41% of DLBCL had PD-L1+ tumor cells 26% of DLBCL had mPD-L1+ ME cells	NA
	Vranic S. & al.,[86] *PLOS one* 2016 (n=49)	PD-L1 IHC FFPE (Ventana / SP142) PD-L1 IHC FFPE (Ventana / SP263)	PD-L1+ if >5% cells had a 2+ or 3+ level of staining intensity	NA	50% of rDLBCL had PD-L1+ (SP142) tumor cells 57% of rDLBCL had PD-L1+ (SP263) tumor cells	NA
	Kwon D. & al.,[59] *Histopathology* 2016 (n=126)	PD-L1 IHC FFPE (Cell signaling / E1L3N)	PD-L1+ if >10% cells had a 1, 2 or 3 level of staining intensity	NA	61.1% of DLBCL had PD-L1+ (ABC>GCB) tumor cells	No significant difference between PD-L1^+^ and PD-L1^-^ DLBCL (*p=0.238*) (univariate analysis)
	Menter T. & al.,[68] *Hum Pathol* 2016 (n=260)	PD-L1 IHC FFPE (Cell signaling / E1L3N)	PD-L1+ if >5% cells showed positive staining	NA	31% of DLBCL had PD-L1+ tumor cells	NA (done for HL)
FL	Carreras J. & al.,[78] *JCO* 2009 (n=5)	PD-L1 Flow cytometry (Pharmingen / MIH1)	If cells showed greater PD-L1+ staining relative to the isotype control	NA	FL tumor cells were PD-L1-	NA (done for PD-1)
	Laurent C. & al.,[10] *Oncoimmunol*. 2015 (n=27)	PD-L1 IHC FFPE (Ventana / SP142)	PD-L1+ if >10% cells had a 2+ or 3+ level of staining intensity	mPD-L1+ if >10% cells had a 2+ or 3+ level of staining intensity	FL tumor cells were PD-L1- 10% of FL had mPD-L1+ ME cells	NA
	Menter T. & al.,[68] *Hum Pathol* 2016 (n=59)	IHC FFPE (Cell Signaling / E1L3N)	PD-L1+ if >5% cells showed positive staining	NA	6% of grade 1-2 FL and 11% of grade 3 FL were PD-L1+ (weak expression)	NA (done for HL)
	Ramsay AG. & al.,[83] *Blood* 2012 (n=59)	PD-L1 IHC FFPE (Abcam / polyclonal)	NA	NA	Intrafollicular FL cells were PD-L1+	Increased PD-L1 expression in the FL poor-prognosis group (survival < 5 yrs) vs in the FL good-prognosis group (survival > 15 yrs) (univariate analysis)
PMBL	Andorsky D. & al.,[12] *CCR* 2011 (n=3)	PD-L1 IHC frozen (eBioscience / MIH1)	NA	NA	100% of PMBL are PD-L1+ (undefined cell type)	NA
	Chen B. & al.,[13] *CCR* 2013 (n=21)	PD-L1 IHC FFPE (Sino Bio / 15)	PD-L1+ if >5% cells had a 2+ or 3+ level of staining intensity	mPD-L1+ if >20% ME (tissue area) had a 2+ or 3+ level of staining intensity	71% of PMBL had PD-L1+ tumor cells 90% of PMBL had PD-L1+ ME cells	NA
	Vranic S. & al.,[86] *PLOS one* 2016 (n=3)	PD-L1 IHC FFPE (Ventana / SP142) PD-L1 IHC FFPE (Ventana / SP263)	PD-L1+ if >5% cells had a 2+ or 3+ level of staining intensity	NA	100% of rPMBL had PD-L1+ (SP142) tumor cells 100% of rPMBL had PD-L1+ (SP263) tumor cells	NA
PL	Laurent C. & al.,[58] *Haematologica* 2015 (n=40)	PD-L1 IHC FFPE (Ventana / SP142)	PD-L1+ if >5% cells had a 2+ or 3+ level of staining intensity	PD-L1+ if >10% cells had a 2+ or 3+ level of staining intensity	2.5% of PL had PD-L1+ tumor cells 60% of PL had PD-L1+ ME cells 78% of EBV+ PL had PD-L1+ tumor cells	NA (done for EBV)
CLL	Xerri L. & al.,[56] *Hum Pathol* 2008 (n=11)	PD-L1 flow cytometry (mice immunization / PD-L1.3. 1)	PD-L1+ if >1% cells had a 1+, 2+ or 3+ level of staining intensity	NA	0% of CLL had PD-L1+ tumor cells (blood)	NA
	Brusa D. & al.,[84] *Haematologica* 2014 (n=20)	PD-L1 IHC FFPE (Novus biologicals / polyclonal)	% of PD-L1+ areas in proliferative centers (PC)	NA	10% of CLL cells had PD-L1+ PC vs 5% outside PC	NA
	Ramsay AG. & al.,[83] *Blood* 2012 (n=35 for clinics, n=71 for IHC)	PD-L1 IHC FFPE (Abcam / polyclonal) Flow cytometry (eBioscience / MIH1)	NA	NA	Increased expression of PD-L1 in CD20+ B cells from CLL lymph nodes and blood (vs healthy donors).	Increased PD-L1 expression in the CLL poor-prognosis group (survival < 5 yrs) vs in the CLL good-prognosis group (survival > 15 yrs). (univariate analysis)
	Menter T. & al.,[68] *Hum Pathol* 2016 (n=37)	PD-L1 IHC FFPE (Cell signaling / E1L3N)	PD-L1+ if >5% cells showed positive staining	NA	3% of CLL had PD-L1+ tumor cells	NA (done for HL)
T-NHL	Wilcox R.A. & al.,[85] *Blood* 2009 (n=54 PTCL; n=20 AITL; n=8 CTCL)	PD-L1 IHC FFPE (NA / 5H1) PD-L1 IHC FFPE (NA / 5H2) PD-L1 IHC FFPE (NA / 5H3)	PD-L1+ if >30% cells showed positive staining	NA	17% of PTCL had PD-L1+ tumor cells 5% of AITL had PD-L1+ tumor cells 27% of CTCL had PD-L1+ tumor cells	NA
	Andorsky D. & al.,[12] *CCR* 2011 (n=5)	PD-L1 Flow cytometry frozen (eBioscience / MIH1)	NA	NA	80% of ALCL are PD-L1+ (undefined cell type)	NA
	Brown J.A. & al.,[87] *JI* 2003 (n=11)	PD-L1 IHC FFPE (mice immunization / Ab29E.2A3)	If cells showed greater PD-L1+ staining relative to the isotype control	NA	64% of PTCL are PD-L1+ (undefined cell type)	NA
	Miyoshi H. & al.,[92] *Blood* 2016 (n=135)	PD-L1 IHC FFPE (Abcam / EPR1161)	PD-L1+ if >50% cells showed positive staining	mPD-L1+ if > 10 cells PD-1+ / HPF	7% of ATLL had PD-L1+ tumor cells 59% of ATLL had PD-L1+ ME cells	PD-L1+ ATLL (expressed on tumor cells) have a worse OS compared to PD-L1- ATLL (40% vs 10% at 2 yrs, *p=0.0085*). PD-L1+ ATLL (on ME cells) have a better OS compared to PD-L1- ATLL (48% vs 20% at 2 yrs, *p=0.0029*) (maintained in multivariate analysis with Adj *p=0.0322* and Adj *p=0.0014*, respectively)
	Vranic S. & al.,[86] *PLOS* *one* 2016 (n=11)	PD-L1 IHC FFPE (Ventana / SP142) PD-L1 IHC FFPE (Ventana / SP263)	PD-L1+ if >5% cells had a 2+ or 3+ level of staining intensity	NA	28% of rPTCL had PD-L1+ (SP142) tumor cells 28% of rPTCL had PD-L1+ (SP263) tumor cells	NA
**PD-1 protein expression in NHL**						
DLBCL	Ko Y. & al.,[60] *Korean J Pathol* 2011 (n=63)	PD-1 IHC FFPE (Abcam / NAT)	NA	mPD-1+ if >20 TILs PD-1+ / HPF	52.4% of DLBCL had mPD-1+ ME cells	mPD-1+ was associated with a poor prognosis in DLBCL patients (*p=0.12*, univariate analysis)
	Ahearne M. & al.,[70] *Virchows Arch* 2014 (n=70)	PD-1 IHC FFPE (Dr Roncador / NA)	NA	mPD-1+ > Median expression of PD-1	PD-1+ cells were found in rosettes around lymphoma cells	High PD-1 expression correlated with OS (95% vs 60% at 2 years p=0,0007) (maintained in multivariate analysis with Adj *p=<0.05*)
	Muenst S. & al.,[55] *Disease Markers* 2010 (n=55 for clinics / n=184 for IHC)	PD-1 IHC FFPE (R&D, polyclonal)	NA	mPD-1+ > 2.8% cells PD-1+	PD-1+ TILs found in transformed DLBCL were more abundant than those found in primary DLBCL	mPD-1+ TILs showed a prognostic significance for DSS (100% vs 80% at 2 years, p=0.032, univariate analysis) (not maintained in multivariate analysis with *Adj p>0.05*)
	Zhang W. & al.,[73] *Cancer Medicine* 2016 (n=50 for clinics / n=31 for IHC)	PD-1 IHC FFPE (Abcam / NAT) PD-1 flow cytometry (BD Biosciences / MIH4)	H score calculation: H = (PD-1 staining intensity: 0, 1, 2, 3) x (percentage of positive cells: 0 for <1%, 1 for 1-25%, 2 for 25-49%, 3 for >50%)	mPD-1+ CD4+ if > 30.25% of cells showed positive staining	65% of DLBCL were PD-1+ (H>1) (undefined cell type) No correlation between % of PD-1+/CD4+ in blood vs % of PD-1+ cells in tissues	EFS and OS were lower in PD-1+ patients (*p>0.05*, not significant) Patients with higher % mPD-1+CD4+ T cells had lower EFS and OS (90% vs 50% at 15 months *p=0.005*; 97% vs 65% at 15 months *p=0.009*, respectively, univariate analysis)
	Kwon D. & al.,[59] *Histopathology* 2016 (n=78 for clinics / 121 for IHC)	PD-1 IHC FFPE (Cell Marque / MRQ-22)	NA	Number of PD-1+ cells / HPF (Groups: 1 (<10), 2 (10-30), 3 (>30))	68.6% of DLBCL had PD-1+ TILs	A high number of PD-1+ TILs correlated with a high OS (80% vs 55% at 2 years, *p=0.026*) and high PFS (80% vs 40% at years, *p=0.005*) in R-CHOP-treated DLBCL patients. (maintained in multivariate analysis for OS with Adj *p=0.006*)
	Laurent C. & al.,[58] *Oncoimmunol* 2015 (n=27)	PD-1 IHC FFPE (Abcam / NAT105)	PD-1+ if >60% cells had a 2+ or 3+ level of staining intensity	PD-1+ if >10% cells had a 2+ or 3+ level of staining intensity	22% of DLBCL had PD-1+ tumor cells 48% of DLBCL had PD-1+ ME cells	NA
FL	Carreras J. & al.,[78] *JCO* 2009 (n=89 for clinics / n=100 for IHC)	PD-1 IHC FFPE (Abcam / NAT-105)	NA	NA	21,8% of FL cells are PD-1+ (undefined cell type)	A high number of PD1+ cells was associated with a high PFS and OS (75% vs 50% at 2 years, *p=0.038*; 100% vs 80% at 2 years, *p=0.004*, respectively) (maintained in multivariate analysis for OS with Adj *p=0.013*)
	Richendollar BG. & al.,[79] *Hum* *Pathol* 2011 (n=91)	PD-1 IHC FFPE (Abcam / NAT-105)	NA	PD-1+ if > 35.6 PD-1+ cells / HPF	49% of FL had intrafollicular PD-1+ cells (undefined cell type)	A high number of mPD-1+ T cells was associated with a decreased OS (*p=0.10*, not significant, univariate analysis)
	Smeltzer J. & al.,[81] *CCR* 2014 (n=58)	PD-1 IHC FFPE (Abcam / NAT-105)	Follicular vs diffuse pattern	Follicular vs diffuse pattern	FL were PD-1+, with diffuse or intrafollicular pattern of expression. Pattern of PD-1+ expression predicted clinical outcome.	Diffuse PD-1 expression was associated with a shorter time to transformation, or TTT (*p=0.033*) and inferior OS (85% vs 45% at 5 years, *p=0.009*). (maintained in multivariate analysis with Adj *p=0.045* for TTT, Adj. *p=0.012* for OS)
	Wahlin BE. & al.,[75] *CCR* 2010 (n=64)	IHC FFPE (CNIO gift / NA)	Follicular vs interfollicular pattern	Follicular vs interfollicular pattern	PD-1 expression was more frequent inside than outside the follicles in FL.	Follicular PD-1 expression was associated with a good outcome (multivariate analysis with Adj. *p=0.0689*)
	Yang ZZ. & al.,[82] *Blood Cancer Journal* 2015 (n=32)	Flow cytometry (NA / NA)	NA	Dimly or brightly staining for PD-1 expression in T CD4+ or CD8+ cells,	PD-1 was expressed at high and low levels in CD4+ and CD8+ T cells	CD4+ mPD1+dim was associated with poorer OS (95% vs 75% at 5 years, *p=0.007*, univariate analysis) CD8+ mPD-1dim was associated with poorer OS (100% vs 70% at 5 years, *p=0.026* univariate analysis)
	Ramsay AG. & al.,[83] *Blood* 2012 (n=59)	PD-1 IHC FFPE (Abcam / NAT-105)	NA	NA	Increased expression of PD-1 in FL interfollicular T cells (vs reactive tissues)	Increased PD-1 expression in the FL poor-prognosis group (survival < 5 yrs) vs in the FL good prognosis group (survival > 15 yrs) (Univariate analysis)
	Dorfman D. & al.,[77] *Am J Surg Pathol* 2006 (n=6)	PD-1 IHC FFPE (mice immunization / EH12)	NA	mPD-1+ if > 20% cells showed positive staining	0% of FL had PD-1+ tumor cells	NA
PL	Laurent C. & al.,[58] *Haematologica* 2016 (n=40)	PD-1 IHC FFPE (Abcam / NAT-105)	PD-1+ if >5% cells had a 2+ or 3+ level of staining intensity	PD-1+ if >10% cells had a 2+ or 3+ level of staining intensity	5% of PL had PD-1+ tumor cells 60% of PL had PD-1+ ME cells 54% of EBV+ PL had PD-1+ ME cells	NA (done for EBV)
CLL	Xerri L. & al.,[56] *Hum Pathol* 2008 (n=11)	PD-1 flow cytometry (mice immunization / PD-L1.3. 1)	PD-L1+ if >1% cells had a 1+, 2+ or 3+ level of staining intensity	NA	91% of CLL had PD-1+ tumor cells	NA
	Brusa D. & al.,[84] *Haematologica* 2013 (n=20)	PD-1 IHC FFPE (R&D / polyclonal)	NA	% of PD-1+ areas in proliferative centers (PC)	In CLL, PD-1 is expressed at higher levels in PC (13% of PD-1+ areas) compared to other place (8% of PD-1+ areas)	NA
	Ramsay AG. & al.,[83] *Blood* 2012 (n=35 for clinics, n=71 for IHC)	PD-1 IHC FFPE (Abcam / NAT-105) Flow cytometry (eBioscience / MIH4)	NA	NA	PD-1 expression was increased in CLL T cells (vs reactive tissues and healthy donor blood)	Increased PD-1 expression in CD3+ cells in the CLL poor-prognosis group (survival < 38 months) vs in the CLL good prognosis group (survival > 10 yrs) (Univariate analysis)
T-NHL	Cetinözman F. & al.,[88] *Am J Surg Pathol* 2012 (n=11 MF tumor stage; n=26 PCSM-TCL)	PD-1 IHC FFPE (R&D / polyclonal)	If cells showed greater PD-1+ staining relative to the isotype control	NA	9% of MF had PD-1+ tumor cells 100% of PCSM-TCL had PD-1+ tumor cells	NA
	Cetinözman F. & al.,[90] *Arch* *Dermatol* 2012 (n=27 SS: n=22 MF tumor stage)	PD-1 IHC FFPE (R&D / polyclonal)	PD-1+ if >50% cells showed positive staining	NA	89% of SS had PD-1+ tumor cells 14% of MF had PD-1+ tumor cells	NA
	Miyoshi H. & al.,[92] *Blood* 2016 (n=135 ATLL)	PD-1 IHC FFPE (Abcam / NAT-105)	NA	Average number of PD-1+ TILs / HPF	19% of ATLL had PD-1+ tumor cells	NA (done for PD-L1)

NHL subtype, number of cases, antibodies used, scoring method and conclusions (PD-1/PD-L1 expression and its link with prognosis, when available) are specified for each study. FFPE: formalin-fixed and paraffin-embedded; CTCL: cutaneous T cell lymphoma; PC: proliferative center; HPF: high power field; NA: not available.

## THE PD-1/PD-L1/2 AXIS IN DLBCL

Diffuse large B cell lymphoma (DLBCL) are the most common type of lymphoma in adults. The prognosis for DLBCL patients is heterogeneous and remains poor in 40% of cases despite the introduction of therapy combining rituximab with cyclophosphamide-doxorubicin-oncovin-prednisone (CHOP) [44–46]. The analysis of gene expression profiles in DLBCL has allowed the identification of three different DLBCL entities: germinal center B cell-like (GCB), activated B cell-like (ABC), and primary mediastinal B cell-type (PMBL) [47]. These subtypes arise from different stages of B-cell differentiation and acquire distinct oncogenic abnormalities which promote tumor proliferation and survival [47, 48]. The GCB subtype more frequently presents with genetic lesions such as BCL2 translocations, PTEN or ING1 deletions, MDM2 gains or amplifications and p53 mutations. In contrast, DLBCL ABC exhibit chronic BCR activation (*e.g*. through CD79A/B mutations) and present with other genetic alterations such as BCL2 amplifications or INK4-ARF deletions. The chronic BCR signaling pathway is a well-known target for therapeutic interventions (*e.g* ibrutinib, PKC inhibitors, lenalidomide) but activating mutations (*e.g* of CARD11, Bcl10 translocations, A20 deletions) occasionally hamper drug efficacy [48]. However, the physiopathology of DLBCL is not limited to tumor cells since the DLBCL microenvironment (ME) has also proven to be mandatory for its carcinogenesis. Within the ME, the tumor stromal cells and the composition of the immune infiltrate influence the progression of the DLBCL disease [49–52]. In addition, the strength of the immune response can be functionally impaired by several tumor immune escape mechanisms, most notably those upregulating immune checkpoint molecules such as PD-1/PD-L1 [53].

### PD-1/PD-L1/2 expression in DLBCL

**Figure 2:**
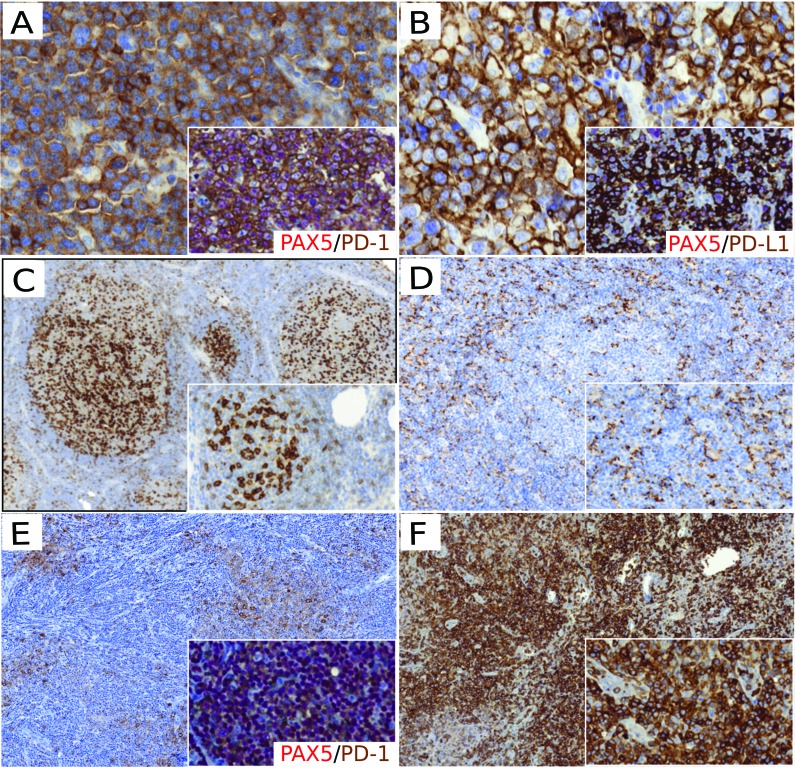
PD-1/PD-L1 protein expression in non Hodgkin lymphoma **A**. PD-1 staining positive in DLBCL tumor cells (x400); the magnified insert showed PD-1^+^ (brown) / PAX5^+^ (red) tumor cells (x400). **B**. PD-L1 staining positive in DLBCL tumor cells (x400); the magnified insert showed PD-L1^+^ (brown) / PAX5^+^ (red) tumor cells (x400). **C**. PD-1 staining showing PD-1^+^ T_FH_ cells with an intrafollicular pattern in FL samples (x100; magnified insert x200). **D**. PD-L1 staining showing PD-L1^+^ macrophages in the FL ME (x100). **E**. PD-1 staining showing PD-1^+^ cells in CLL proliferative centers (x100); the magnified insert showed PD-1^+^ (brown) / PAX5^+^ (red) neoplastic B cells in proliferative center (x200). **F**. PD-1 staining in AITL samples showing PD-1^+^ tumor cells (x200). **D**. PD-L1 staining showing PD-L1 ^+^ macrophages in the FL ME (x100; magnified insert x100). **F**. PD-1 staining in AITL samples showing PD-1 ^+^ tumor cells (x200; magnified insert x400).

PD-L1 is expressed by both DLBCL tumor B cells and by non-malignant cells from their immune microenvironment, such as macrophages [10, 54]. In DLBCL, PD-L1 expression has been reported in around 20-30% of DLBCL cases but this figure varies greatly depending on the cut-off applied (which ranges from 5 to 30%) and the cell compartment analyzed (tumor/non-tumor cells) [10, 12, 13, 54] (Figures 2A and 2B) (Table 1). All of the studies that have investigated PD-L1 levels in DLBCL have reported higher expression rates in the non-GCB DLBCL subtypes [10, 12, 13, 54]. In contrast, the expression of PD-L2 has been less well documented, as most NHL cell lines do not express it [12]. One report found low PD-L2 expression in DLBCL cells without a significant difference between subtypes [10]. Recently, a retrospective study conducted a double staining of PD-L1 and PAX5 in DLBCL samples in order to precisely quantify the rate of PD-L1^+^ cells in both the tumor and non-tumor compartments [54]. They found that 10.5% of DLBCL samples expressed PD-L1 in tumor cells (*n*=132/1253 DLBCL samples; using a cut-off of 30%), while it was expressed in 15.3% of ME cells, which were essentially composed of macrophages (*n*=172/1121 DLBCL samples; using a cut-off of 20%). This study also confirmed the predominant expression of PD-L1 in non-GCB subtypes of DLBCL NOS.

In contrast to PD-L1, PD-1 expression has almost exclusively been detected in the ME cells of DLBCL, with a varying number of cells per mm^2^ examined [10, 55, 56]. DLBCL tumor cells have been found to express a low level of cell surface PD-1 [10, 55–57], and sometimes co-express both PD-1 and PD-L1 [10, 58]. Kiyasu et al. [54] also reported that the number of PD-1^+^ TILs was higher in GCB DLBCL and was inversely correlated with the number of PD-L1^+^ tumor and ME cells, although these conclusions remain controversial [59, 60].

PD-L1 expression is also considered to be a hallmark of EBV-associated lymphoproliferative disorders. These include EBV^+^ plasmablastic lymphoma (PL) (where 20% of tumor cells are PD-L1^+^) [58], EBV^+^ post-transplant lymphoproliferative disorders (PTLD) (where 60% of tumor cells are PD-L1^+^) [13], EBV^+^ DLBCL of the elderly (where 100% of tumor cells are PD-L1^+^) [13] and the recently described EBV^+^ DLBCL subtype [31] found in young patients (where 76% of patients display an expression of PD-L1 by more than 5% of their tumor B cells) [61]. This frequent up-regulation of PD-L1 by EBV^+^ lymphoma cells, and the inhibition of EBV-induced lymphomagenesis following PD-1/PD-L1 blockade in a mouse model [62], suggest a link between EBV infection and PD-1/PD-L1 upregulation. Furthermore, PD-1 and PD-L1 have also been shown to be expressed by infiltrating immune cells of EBV^+^ lymphoma patients, suggesting that their expression underlies the tolerogenic immune response induced by EBV [58, 61]. However, PD-L1 expression does not always correlate with EBV infection since it has also been reported in EBV^-^ PTLD [13] and EBV^-^ PL [13, 58].

### Mechanisms of PD-L1 and PD-L2 overexpression in DLBCL tumor cells

Nearly 20% of DLBCL NOS are reported to carry genetic anomalies and chromosomal alterations that lead to PD-L1/2 overexpression [35]. Specifically, the structural anomalies on chromosome 9p24.1 have been significantly correlated with PD-L1 expression in DLBCL [54, 63]. Other translocations involving *IGH* genes that lead to PD-L1 overexpression have also been reported [35]. Recently, Georgiou *et al*. [35] reported 12% of gains, 3% of amplifications and 4% of translocations of the PD-L1/PD-L2 locus in the non GCB subtype, and lower rates in the GCB subtype. Around 30% of non-GCB DLBCL are also reported to carry MYD88 mutations that cause the chronic activation of the JAK/STAT pathway and in turn stimulate the expression of PD-L1 [35, 39, 42, 43]. Likewise, EBV infection could account for the over-expression of PD-L1 in EBV^+^ DLBCL since antiviral and inflammatory cytokine responses also activate the JAK/STAT pathway. In addition, the EBV-encoded latent membrane protein (LMP)-1 activates AP-1 (*via* cJUN/JUN-B components) and the JAK/STAT signaling pathways which, respectively, activate the PD-L1 enhancer and promoter [38].

Beside DLBCL NOS, primary central nervous system large B cell lymphoma (PCNSL) and primitive testicular lymphoma (PTL) are extranodal DLBCLs that arise at sites considered to be immune sanctuaries [64, 65]. PCNSL and PTL frequently harbor genetic anomalies on chromosome 9p24.1, with 9p24.1 copy gains found in 54% of PTL and 52% of PCNSL [66]. Moreover, translocations involving the PD-L1/L2 locus were also reported in 4% of PTL and 6% of PCNSL [63, 66]. However, further studies of PD-L1 immunostaining with larger cohorts of these rare DLBCL subtypes are needed to confirm this PD-L1 overexpression, as only 10% of PCNSL cases (n=2/20) were found to harbor PD-L1^+^ tumor cells [67].

The expression of PD-L1 by tumor cells in primary mediastinal B cell lymphoma (PMBL) has also been investigated by a number of studies and is reported in 36% to 100% of cases [12, 13, 56, 68]. In PMBL, PD-L1 up-regulation is usually caused by genetic alterations, with 29-55% of chromosome 9p24.1 gains [63, 66] and 20% of rearrangements at the *PD-L1/2* locus, involving either the *CIITA* or the *IGH* loci [63, 69].

### The prognostic impact of PD-1/PD-L1 expression in DLBCL

**Figure 3:**
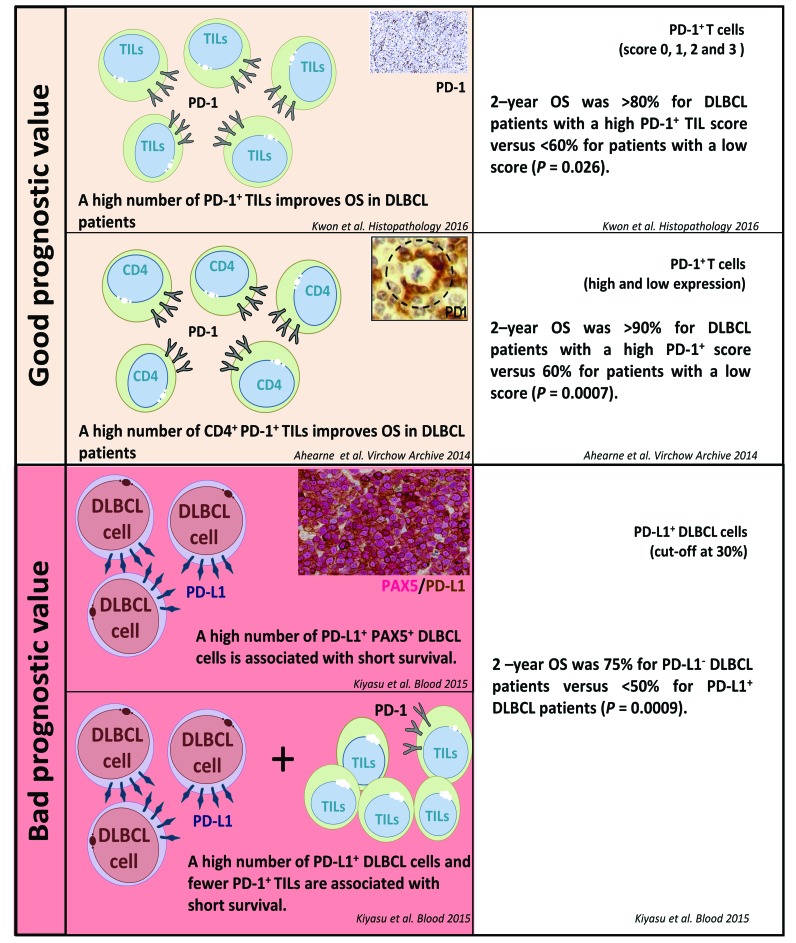
PD-1/PD-L1 expression and their prognostic value in diffuse large B cell lymphoma

As depicted in Figure 3, PD-1 and PD-L1 expression in DLBCL samples have a prognostic value in DLBCL [54, 59, 70]. Using a large series of 1200 DLBCL samples, Kiyasu et al. [54] demonstrated that patients with PD-L1^+^ DLBCL had inferior overall survival rates than PD-L1^-^ DLBCL patients. Moreover, patients with PD-L1^+^ tumor cells but low PD-1^+^ TIL counts had poorer prognoses than patients with PD-L1^-^ DLBCL and high PD-1^+^ TIL counts [54]. Elevated soluble plasma PD-L1 (sPD-L1) levels in DLBCL patients has also been shown to correlate with the lowest three-year overall survival rates (3-year OS of 76% versus 89%, P<0.001) [71, 72]. Moreover, reports of tumor cell PD-L1 expression have been almost completely assigned to the non-GC subtype of DLBCL which have the worst prognosis among the DLBCL subtypes [10, 12, 54]. Other studies have correlated better DLBCL patient survival with their TILs having a higher PD-1 expression [55, 59, 70]. Conversely, however, DLBCL patients with a high PD-1 expression on their circulating CD4^+^ T cells showed an aggressive clinical course [73].

## THE PD-1/PD-L1 AXIS IN FL

### Expression of the PD-1/PD-L1/2 axis in FL

In contrast to DLBCL, most follicular lymphoma (FL) tumor cells do not express PD-L1 or PD-L2 [10, 12, 68, 74], however PD-1^+^ cells are abundant in the ME of FL [10]. In FL, these PD-1^+^ cells include not only TILs but also follicular helper T cells (T_FH_) from lymphomatous follicles or residual germinal centers [75, 76] (Figure 2C). FL ME macrophage cells have an increased expression of PD-L1 (Figure 2D), but these levels remain lower than those in the ME of DLBCL [10].

### The prognostic impact of PD-1/PD-L1 expression in FL

**Figure 4:**
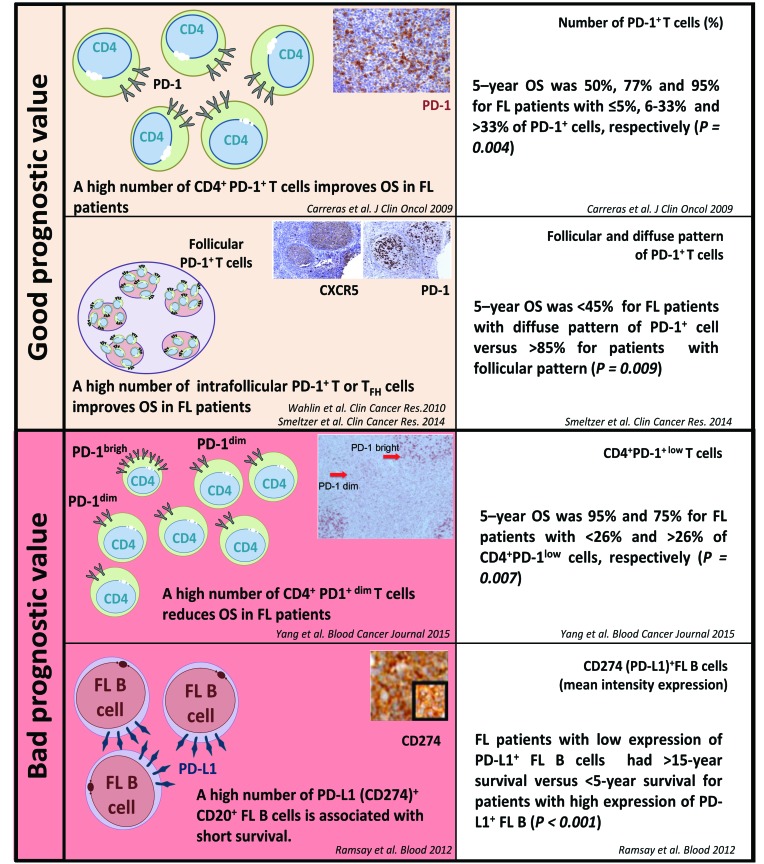
PD-1/PD-L1 expression and their prognostic value in follicular lymphoma

Conflicting data have been reported regarding the prognostic impact of PD-1/PD-L1 expression in FL. As depicted in Figure 4, some studies have reported a correlation between low PD-1^+^ cell counts and either a high histological grade of FL or a higher risk of transformation to DLBCL [77]. Carreras et al. showed an association between high PD-1^+^ cell counts and better overall survival (OS) and progression-free survival (PFS), although these results remain controversial [78]. These divergent results could possibly be accounted for by the various subtypes of PD-1^+^ lymphocytes tested (T_FH_, exhausted T cells, or Tregs). The prognostic value of PD-1 staining in immune cells may also rely on the pattern of infiltration in FL samples, since high rates of intra-follicular PD-1^+^ cells have been correlated with a good prognosis while PD-1^+^ inter-follicular and diffuse infiltrates have poorer outcomes [75, 79–81]. In addition, flow cytometry measures of PD-1 expression levels evidenced two different CD4^+^ PD-1^bright^ and CD4^+^ PD-1^dim^ T cell subtypes that were associated with different outcomes [82]. In contrast to PD-1, the prognostic relevance of PD-L1 staining in FL remains poorly studied so far. Only one study reported a significant correlation between PD-L1 expression by tumor cells and lower survival rates of FL patients [83].

## THE PD-1/PD-L1/2 AXIS IN OTHER SMALL B CELL LYMPHOMA

The PD-1/PD-L1 axis also remains poorly documented in other small B cell lymphoma. From the few available studies it appears that LPL, MCL and MZL tumor cells are usually negative for PD-L1 IHC staining [12, 56, 68]. In CLL, PD-1 is expressed by both reactive T cells and some paraimmunoblasts and prolymphocytes in the proliferative centers [56, 84] (Figure 2E). Few studies have reported PD-L1^+^ cells among the circulating blood cells of CLL patients [83, 84], and the expression of PD-L1/L2 by CLL tumor cells showed conflicting results [12, 56, 68, 84].

## THE PD-1/PD-L1/2 AXIS IN T CELL LYMPHOMA

The PD-1/PD-L1 axis is involved in both the development and the immune escape of some PTCL malignancies. From a diagnostic point of view, PD-1 expression is usually observed at the surface of angioimmunoblastic T cell lymphoma (AITL) tumor cells derived from normal T_FH_. This also applies to other lymphoma with a TFH phenotype according to the new WHO 2016 classification [31] including follicular T cell lymphoma (FTL) and other PTCL with a TFH phenotype (PTCL-TFH) (Figure 2F). PD-1/PD-L1 expression has also been observed on the surface of tumor ME immune cells in these PTCL subtypes, however PD-L1 expression has rarely been observed in tumor cells from these T_FH_ subtypes, with no PD-L1^+^ tumors from 4 cases reported in one study [56] and 1/21 elsewhere [85]. PD-L1 expression appears to vary widely amongst tumor cells, with low rates ranging from 0% [56] to 17% of cases [85], however one study has suggested that it is higher in refractory PTCL NOS, with 28% of rPTCL NOS cases being PD-L1^+^ [86].

In contrast, Marzec *et al*. [41] reported a consistent overexpression of PD-L1 in anaplastic lymphoma kinase-positive anaplastic large cell lymphoma (ALK^+^ ALCL) cell lines. This report was confirmed in biopsies from ALK^+^ ALCL tumors, with frequencies of PD-L1^+^ cases varying from 34 to 100% of the analyzed cases [12, 85, 87]. Marzec et al. [41] showed that activation of the transcription factor STAT3 by the nucleophosmin-ALK (NPM-ALK) fusion protein could be responsible for the increased expression of PD-L1 at the cell surface of ALK^+^ tumor cells. Moreover, the same group showed that NPM-ALK induces the activation of the IL-10 and TGF-β cytokines. Since IL-10 can also activate JAK/STAT signaling *via* STAT3, and thus induce the up-regulation of PD-L1 [39], one may reasonably speculate that NPM-ALK directly up-regulates PD-L1 *via* STAT3 or IL-10.

The expression of PD-1 and/or PD-L1 has also been reported in some cutaneous lymphoproliferative disorders. For instance, some mycosis fungoïdes (MF) and Sezary syndrome (SS) tumor cells have been found to express PD-1 at their surface. Similarly, some rare cutaneous disorders characterized by small- and average-sized CD4^+^ T cells have been found to be PD-1^+^ [88–90]. In addition, PD-L1 expression has been reported on SS/MF tumor cells and on SS/MF tumor-infiltrating dendritic cells in 27% and 73% of cases, respectively [85]. Likewise, cases of HTLV1-associated leukemia and lymphoma of adults (ATLL) have been shown to overexpress PD-L1 at the surface of their tumor or ME stromal cells [54, 91, 92], and the presence of PD-L1^+^ tumor cells, together with the lack of PD-L1^+^ ME cells, has been correlated with a poor prognosis in ATLL patients [92].

Finally, studies on extranodal NK/T cell lymphoma-nasal type have reported PD-L1^+^ tumor cells in 67% of cases. This was presumably secondary to EBV infection *via* LMP1-mediated upregulation of PD-L1 or *via* interferon (IFN) signaling [13, 43].

## CLINICAL SIGNIFICANCE OF STUDYING PD-1/PD-L1/2 EXPRESSION IN NHL PATIENTS

Preliminary data from clinical trials in solid tumors and HL indicate that patients with PD-L1^+^ tumor cells were those who benefitted most from PD-1/PD-L1 immune checkpoint blockade treatments [15, 93–94]. Nevertheless, significant responses were also observed in some of the PD-L1^-^ patients [95]. This observation reflects the limits of using PD-L1 protein expression alone as a single predictive biomarker since its expression is heterogeneous among tumor cells and can increase spontaneously or upon treatment. The cell-to-cell heterogeneity of PD-1, PD-L1 and PD-L2 expression among cells from NHL biopsies can now be assessed by single cell RNA-sequencing technologies, but these are not widely available in diagnostic laboratories so the current clinical need is for an IHC-based test to detect cell surface protein markers. This has its own problems, however, since there is a lack of standardized IHC procedures [96] with different antibody clones and detection thresholds [97]. In fact, the levels of PD-1 and PD-L1 protein expression and their impact on clinical response in NHL patients do differ according to lymphoma subtype and the staining assessment methodology (Table 1). In solid tumors recent evidence has suggested that both mutational load, preexisting CD8^+^ T cell infiltration and PD-L1 expression (with a 1% cut-off) represent predictive factors for immunotherapeutic response [94, 98]. So far, none of these biomarkers have been validated in NHL, although we and others have reported significant levels of PD-L1 expression in these malignancies. However, preclinical trials with immune checkpoint blockade (ICB) therapies have shown a promising efficacy, especially in relapsed/refractory (r/r) NHL with an acceptable safety profile of toxicity [99]. For example, 36% of r/r DLBCL patients and 40% of r/r FL displayed a complete response (CR) or partial response (PR) with nivolumab [100]. Pembrolizumab had an overall response rate (ORR) of 37.5% in PMBL patients and 21% in CLL patients [101]. In contrast though, TCL patients treated with nivolumab responded at a lower rate, and an overall response was achieved in only 17% of patients [99, 100]. In addition, EBV^+^ lymphoma such as EBV^+^ DLBCL or NKTCL [13, 54, 58, 102] and EBV^+^ or EBV^-^ PTLD frequently show PD-L1-expressing tumor and ME cells. Thus, in these subtypes, PD-L1 may be used as a biomarker to identify patients who may benefit from anti-PD-1/PD-L1 inhibitors. Thus, the clinical impact of PD-L1 status in EBV^+^ lymphoma and PTLD has not yet been firmly established. Nevertheless, a significant association between PD-L1 expression and poor outcomes has been detected in early-diagnosed NKTCL patients treated with chemotherapy containing asparaginase [54, 102]. ALK^+^ ALCL patients may also be eligible for treatment by PD-1/PD-L1 inhibitors since their NPM-ALK rearrangement up-regulates PD-L1 expression. Nevertheless, correlating this expression with clinical outcome needs to be formally validated in ALK^+^ ALCL patients. Ongoing preclinical trials and translational research into the PD-1/PD-L1 axis in lymphoma will assess the effectiveness of PD-1/PD-L1 inhibitors in PD-1/PD-L1-positive r/r B cell and T cell lymphoma. Thus, the validation of standardized procedures for assessing PD-1/PD-L1 expression is paramount for establishing a reliable predictive marker of response, which is currently missing from our therapeutic armament for NHL patients.

Encouraged by the promising results in melanoma [103] and multiple myeloma patients [104], we anticipate that r/r NHL patients will also benefit from ICB therapies combined with other immunotherapeutic agents (such as anti-CD20 or anti-CD30), or from a combination of multiple ICB therapies (such as those targeting PD-1, CD137 and LAG3). Indeed, although the combination of anti-PD-1 with anti-CTLA-4 did not show a greater clinical response than anti-PD-1 alone for the treatment of hematological malignancies [105], many other combinations of ICB therapy are currently under investigation in NHL patients [99].

In conclusion, tumors from most NHLs carry PD-1^+^ and PD-L1/L2^+^ cells. This encompasses both PD-1-expressing TILs as well as PD-L1/2-expressing macrophages and tumor cells in some NHL subtypes. Although PD-1 expression by TILs is the mere hallmark of their physiological activation, PD-L1/2 expression by malignant cells results from several tumor-intrinsic (genetic or oncogenic) and -extrinsic immune escape mechanisms selected by pressure from antitumor immunity. Hence PD-L1 overexpression is associated with the poorest prognosis in several types of aggressive NHLs. Thus, monoclonal antibodies selectively blocking the PD-1/PD-L1 axis could preserve TILs from exhaustion and promote antitumor immunity as an effective therapeutic strategy for NHL.

## Abbreviations

Treg: regulatory T cell
TAM: tumor-associated macrophage
MDSC: myeloid-derived suppressor cell
PD-1: programmed death 1 receptor
PD-L1/2: programmed death ligands 1 and 2
TIL: tumor-infiltrated lymphocyte
FL: follicular lymphoma
NHL: non Hodgkin lymphoma
HL: Hodgkin lymphoma
NK: natural killer cells
PTCL: peripheral T cell lymphoma
TFH: follicular helper T cells
LPL: lymphoplasmacytic lymphoma
MCL: mantle cell lymphoma
MZL: marginal zone lymphoma
CLL: chronic lymphocytic leukemia
DLBCL: diffuse large B cell lymphoma
ME: microenvironment
NOS: not otherwise specified
EBV: Epstein-Barr virus
IGH: immunoglobulin heavy chain
PL: plasmablastic lymphoma
PTLD: post-transplant lymphoproliferative disorder
LMP: latent membrane protein
PCNSL: primary central nervous system large B cell lymphoma
PTL: primitive testicular lymphoma
PMBL: primary mediastinal large B cell lymphoma
OS: overall survival
PFS: progression-free survival
AITL: angioimmunoblastic T cell lymphoma
FTL: follicular T cell lymphoma
ALK: anaplastic lymphoma kinase
ALCL: anaplastic large cell lymphoma
NPM: nucleophosmin
MF: mycosis fungoïdes
SS: Sezary syndrome
ATLL: HTLV1-associated leukemia and lymphoma of adults
IFN: interferon
ICB: immune checkpoint blockade
CR: complete response
PR: partial response
IHC: immunohistochemistry
